# Wearable Sensors Based on Miniaturized High-Performance Hybrid Nanogenerator for Medical Health Monitoring

**DOI:** 10.3390/bios14080361

**Published:** 2024-07-25

**Authors:** Jinjing Wu, Xiaobo Lin, Chengkai Yang, Sirui Yang, Chenning Liu, Yuanyuan Cao

**Affiliations:** 1 Department of Toxicology and Sanitary Chemistry, School of Public Health, Capital Medical University, Beijing 100069, China; 18618306665@mail.ccmu.edu.cn; 2 College of Physics and Optoelectronic Engineering, Shenzhen University, Shenzhen 518060, China; 3 School of Basic Medical Sciences, Capital Medical University, Beijing 100069, China

**Keywords:** triboelectric–electromagnetic hybrid nanogenerator, wearable sensor, mechanical vibration energy harvesting, self-powered, health monitoring

## Abstract

Wearable sensors are important components, converting mechanical vibration energy into electrical signals or other forms of output, which are widely used in healthcare, disaster warning, and transportation. However, the reliance on batteries limits the portability of wearable sensors and hinders their application in the field of Internet of Things. To solve this problem, we designed a miniaturized high-performance hybrid nanogenerator (MHP-HNG), which combined the functions of triboelectric sensing and electromagnetic power generation as well as the advantages of miniaturization. By optimizing the design of TENG and EMG, the wearable sensor achieved a voltage output of 14.14 V and a power output of 49 mW. Based on the wireless optical communication and wireless communication technologies, the wearable sensor achieved the integration of sensing, communication, and self-powered function, which is expected to realize health monitoring, emergency warning, and rehabilitation assistance, and further extend the potential application value in the medical field.

## 1. Introduction

With the popularity of portable electronic products and the continuous development of Internet of Things technology, wearable electronic devices are developed in the direction of intelligence [1]. Nowadays, wearable electronic devices are used in the medical field for positioning and monitoring [2,3,4]. Accurate monitoring of motor status is essential for human health assessment, especially for early diagnosis, early treatment, and medical care rehabilitation [5,6]. It has been reported that one-third of community-dwelling older adults experience at least one fall incident within a year [7]. These accidental falls in the elderly can lead to severe functional decline and even organic lesions, which seriously affect the life quality of the individuals [8]. In addition, to achieve better rehabilitation effects of some chronic diseases, such as stroke, osteoarthritis, and Parkinson’s disease, the patient’s motion states need to be continuously monitored and recorded [9,10,11]. Therefore, real-time health monitoring of the elderly is very important. It is even more critical to provide timely alerts and assistance after falling, aiming to reduce the risk of accidents.

Wearable sensors, as an important part of smart electronic devices, can monitor the physical signals of the human body in real time and realize the efficient sensing of information. However, the problems of high battery demand, frequent replacement of batteries, and excessive waste of batteries producing environmental pollution cannot be ignored [4,12,13,14,15,16,17]. Therefore, it is important to focus on using sustainable, low-cost, and self-powered ways to obtain useful resources from the surrounding environment to adapt to the development of the green information era [4]. As one of the most abundant energies in our living environment, mechanical energy has the features of continuity, easy access, and widespread existence [18]. However, the random motions of the human body are characterized by low frequency, large amplitude, and continuous fluctuations over time [19]. One major challenge to collect energy from low-frequency vibrations is to design a resonant energy harvester to cooperate efficiently with human motions [20,21]. To solve this problem, non-resonant, nonlinear energy-harvesting techniques could be used at low frequency and under low-amplitude excitations [22]. The different kinds of energy conversion mechanisms of nanogenerators have unique working principles and properties, which have been developed to harvest various mechanical vibration energies [23]. Based on the coupling effect of contact charge and electrostatic induction, a triboelectric nanogenerator (TENG) has a high-voltage output, which can effectively integrate self-powered and sensing functions [24,25,26]. Taking advantage of TENG’s excellent sensing properties, motion sensors are capable of detecting mechanical vibration energy and converting it into electrical signals [27]. It can directly reflect the human’s motion, physiological, and pathological states by motion parameters, such as displacements, velocities, and accelerations. However, the traditional TENG is still limited by low current and high impedance, which hinders its further development in the field of sensor applications [24]. One method is improving the performance of TENG by using advanced materials and advanced designs to increase the output power [2,4,22,28]. Hybridizing multiple energy conversion mechanisms into an energy-harvesting system is another approach to obtain more power from a single mechanical movement [29,30]. By integrating the excellent high-current output performance of electromagnetic generators (EMGs), the triboelectric and electromagnetic hybrid nanogenerators (TEHNGs) will enhance the output power and realize functional integration or complementarity [20,23,24,31]. Therefore, the ability to effectively integrate functions and increase the output efficiency is a prerequisite for accurate monitoring [25]. A wearable sensor based on hybrid nanogenerators designed in one study needs to be carried in a backpack because of its large size and weight [32]. Although it increased the output efficiency by simultaneously generating hybrid electrical output under the same mechanical excitation, it is not friendly to the elderly [22]. Many sensors based on different mechanisms have been reported in the medical field (Table S1) [24,32,33,34]. However, it remains a challenge to achieve high-performance output and comfort. Therefore, it is essential to develop miniaturized wearable devices and optimize structures of TEHNG to improve the collection efficiency and provide more power for medical monitoring.

Herein, a miniaturized high-performance hybrid nanogenerator (MHP-HNG) was innovatively designed. The MHP-HNG can realize the functions of integrating effective sensing and efficient energy harvesting by coupling the motion forms of the TENG and EMG to form the same-frequency vibration. The MHP-HNG utilizes magnets to form a flexible structure, which provides repulsive forces to support the mass block. At the same time, the MHP-HNG drives the mass block to produce relative motion in the vertical direction by detecting changes in external mechanical energy. This process provides the necessary conditions for triboelectric sensing and electromagnetic generation. Afterwards, a free-standing grid structure and a Halbach magnet array were designed to optimize the MHP-HNG to improve the sensing signal quality of the TENG while enhancing the magnetic flux density and energy-harvesting efficiency of the EMG, respectively. With an external 1 kΩ resistor, the wearable sensor achieved a peak power output of 49 mW. When the human fell, the wearable sensor reached a sensing voltage output of 14.14 V. Combined with wireless communication technology, the MHP-HNG is not only an excellent energy harvester to power the wearable sensor, but it can also realize remote real-time monitoring of human motion. This might be promising to expand the application of wireless sensing systems and promote their development in the medical field.

## 2. Materials and Methods

### 2.1. Device Information

A digital oscilloscope (Agitek Test Equipment Co. Ltd., Xi’an, China, ZLG, ZDS2024B Plus) equipped with a 100 MΩ impedance probe was utilized for output voltage testing. The short-circuit current was measured by using a low-noise current preamplifier (Stanford, Sunnyvale, CA, USA, SR570). A servo motor ((Leadshine Technology Co. ltd. Shenzhen, Guangzhou, China, SMC600-BAS) was employed to construct a test platform for conducting speed tests. The magnetic field strength of the magnet was measured by using a high-precision Gaussmeter (YiSheng Victor Tech Co. Ltd., Shenzhen, China, VC862A, VICTOR), and inductance strength was assessed by using a multimeter (Fluke Test Equipment Co. Ltd., Shenzhen, China, FLUKE 17B+).

### 2.2. Fabrication of MHP-HNG

The entire shell of the nanogenerator was printed by a 3D printer using polylactic acid (PLA) material, of which the size was 60 mm × 40 mm × 10 mm. PI tape was adhered to the outside of the shell to ensure that the sensor was hermetically sealed to isolate the influence of harsh weather conditions and body sweat [35]. In the triboelectric sensing part, a polytetrafluoroethylene (PTFE) film with a thickness of 0.5 mm was used to fabricate the negative electrode material, a nylon film with a thickness of 0.22 mm was used as the positive electrode material, and double-sided conductive copper foil with a thickness of 0.1 mm was used to make electrodes. The electrodes and PTFE film were attached to the inner shell with quick-drying glue as the stator of the TENG, while the nylon film acted independently as the TENG slider. In the electromagnetic power generation part, the EMG was composed of rubidium iron boron magnets and custom coils. A Halbach magnet array consisting of 10 magnets (30 mm × 3 mm × 3 mm) bonded by quick-drying glue was used as the slider of the EMG. The lacquered copper wire was wrapped up into a rectangular coil by a winding machine, which was fixed on the support plate as the stator of the EMG. The inner and outer diameters of the coil with a total number of 2000 turns were 4 mm × 24 mm and 8 mm × 28 mm, respectively, and the height was fixed at 2 mm.

## 3. Results and Discussion

### 3.1. Structure and Working Principle of MHP-HNG

**Figure 1:**
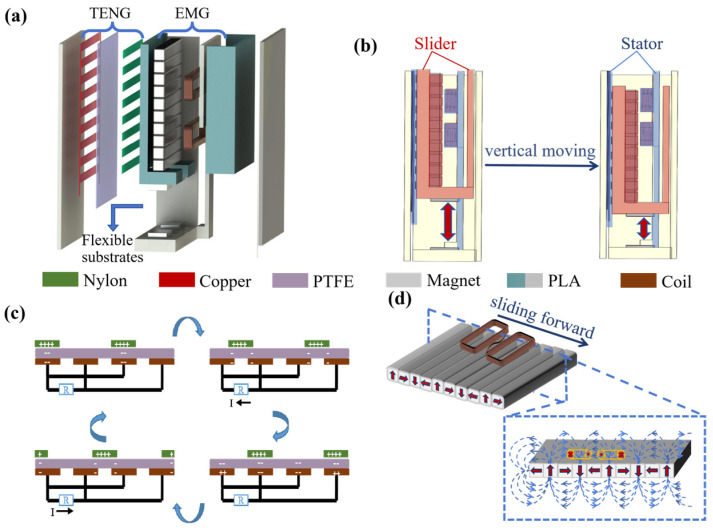
The schematic diagram and working principle diagram of the MHP-HNG. (**a**,**b**) The schematic diagram and vertical motion diagram of the triboelectric and electromagnetic hybrid nanogenerator. (**c**,**d**) The working principle diagram of the TENG and EMG.

The structure of the MHP-HNG is shown in Figure 1a, and the PLA material was prepared by a 3D printer to form the shell of the nanogenerator. The internal structures of the MHP-HNG included the TENG, EMG, and flexible substrates (Figure 1a). As the core part of the sensing function, the TENG consisted of a PTFE film, an electrode with a grid structure, and a nylon film with a grid structure. The PTFE film was selected as a tribo-negative material due to its higher electron-accepting ability compared with other polymer materials [20]. Meanwhile, the PTFE material had a smooth surface, which could effectively reduce the friction damping between TENG layers. The nylon film was used as a tribo-positive material because of its tendency to lose electrons when contacted with PTFE [36]. The EMG consisted of coils and a Halbach magnet array made of 10 neodymium magnets. The stator of the nanogenerator included electrodes attached to the inner shell, PTFE film, and coils glued to the support plate. The stator was fixed on the shell to ensure synchronous movement with the device by quick-drying glue. At the same time, 10 neodymium magnets were attached to the inner wall of the mass block substrate and the nylon film was on the outer wall. Then, the substrate was bonded with a quick-drying glue to form a mass block, which was used as the slider of the nanogenerator, as shown in Figure 1b. The force applied to the whole mass block included the friction between the TENG layers, gravity, and the repulsive forces of the magnet at the bottom. The mass block was in suspension due to repulsive forces between the two magnets at the base of the flexible substrates and at the bottom of the mass block, respectively, which was placed in the opposite direction of the magnetic poles. When the external force on the nanogenerator changed, the repulsive force on the mass block from the bottom magnet would change, the relative movements occurred between the slider and stator, while gravity and friction remained essentially constant. According to the principles of electrostatic induction and triboelectric operation (Figure 1c), the load between the two electrodes generated an alternating current voltage as a triboelectric sensing signal, reflecting the information about the change in speed and acceleration of the human body movement. Meanwhile, relative movements between the Halbach magnet array of the slider and the rectangular coil of the stator caused the magnetic flux change of the coil and generated an electromagnetic power generation signal to power the entire device, as shown in Figure 1d. By collecting the mechanical vibration energy of the human body and converting it into an electrical signal, the wearable sensor based on the MHP-HNG could achieve the integration of responses to human movement and self-powered function.

### 3.2. Optimization of MHP-HNG

**Figure 2:**
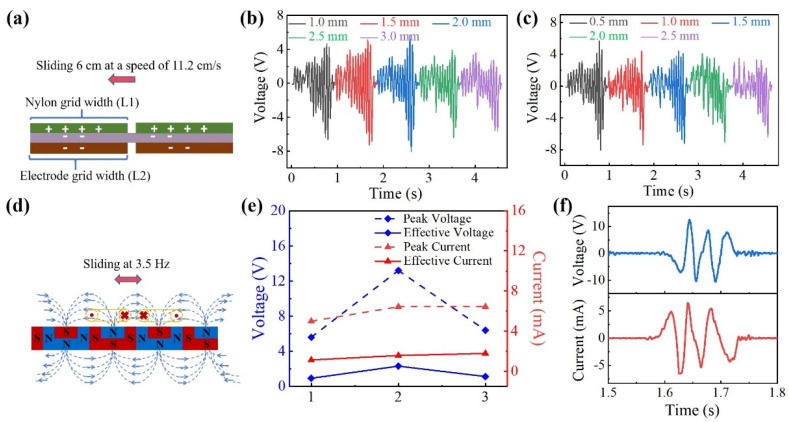
Optimization of the MHP-HNG. (**a**) The schematic diagram of the friction layer of the grid structure. (**b**) Testing the open-circuit voltage output variation of the nylon film grid width (L1) in the range of 1–3 mm. (**c**) Testing the open-circuit voltage output variation of the electrode grid width (L2) in the range of 0.5–2.5 mm. (**d**) The schematic diagram of a series coil. (**e**) Comparing the peak voltage, current, and effective voltage, current output results of three combination modes of single coil (1), series coil (2), and parallel coil (3) at 3.5 Hz are presented. (**f**) Output results of open-circuit voltage output and short-circuit current of the series coil combination under single excitation.

The effect of grid structure width on the sensing performance of the TENG was investigated, aiming to make the wearable sensor better respond to the displacement change information of mass block vibration. The effects of different nylon film grid widths (L1) and electrode grid widths (L2) on the output performance of voltage were tested (Figure 2a). The nylon film and electrodes were cut with laser-marking equipment and the velocity sequence of the friction layer test was provided by the servo motor. To simulate the displacement of the mass block inside the nanogenerator in the vertical direction, the nylon film in the upper friction layer was driven to move against the electrodes and PTFE film in the lower friction layer, sliding 6 cm in a straight line at a speed of 11.2 cm/s (Figure S1). The effect of L1 on the open-circuit voltage output was tested at first. L1 was changed from 1 mm to 3 mm and L2 was fixed to 1 mm. The test results are demonstrated in Figure 2b, where the open-circuit voltage output showed a tendency of increasing and then decreasing at the same sliding speed. When L1 was 2 mm, the open-circuit voltage output reached the maximum value of 8.08 V. Then, the effect of L2 on the open-circuit voltage output was explored, with L1 fixed at 2 mm. The open-circuit voltage output results of L2 are shown in Figure 2c, which ranged from 0.5 mm to 2.5 mm. When the width was 0.5 mm, the open-circuit voltage output reached the maximum value. After that, it showed a downward trend. The reason for this was that when L1 and L2 moved relative to each other, the surface of the nylon and the electrode were gathered on negative and positive charges, respectively. Then, there would be a current inside the TENG. When increasing from the minimum value, the contact area of them would gradually increase to obtain the maximum potential energy and achieve the best output performance at a certain width. If L1 or L2 continued to increase, neighboring electrodes could come into contact, causing a smaller potential difference between neighboring electrodes, which reduced the output performance. These results indicated that a friction layer with a grid structure could respond sensitively to displacement information from linear sliding. Therefore, the optimized L1 and L2 were set to 2.0 mm and 0.5 mm, respectively.

Optimizing the performance of the EMG improved the current output efficiency of nanogenerators. According to the Faraday law of electromagnetic induction and the influences of magnetic flux, the output voltage and magnetic flux can be given by:

(1) $$V=\frac{d\varphi }{dt}*N$$

(2) φ=B∗S

Here, *V* is the induced electromotive force, N is the number of coil turns, and $\frac{d\varphi }{dt}$ is the rate of change of magnetic flux, in Equation (1), while φ is the magnetic flux, B is the magnetic flux density, and S is the area in the vertical direction of the magnetic field, in Equation (2). The output voltage depends on the number of coil turns, and the magnetic flux is proportional to the magnetic flux density.

As for the relationship between the inductance strength and the number of coil turns, it can be expressed as:

(3) $$L=\frac{\mu_{0}*\mu_{r}*N^{2}*A}{I}$$

Here, L is the inductance, $\mu_{0}$ is the permeability in a vacuum, $\mu_{r}$ is the relative permeability of the material in the inner cavity of the coil, N is the number of turns of the coil, A is the cross-sectional area of the coil, and I is the length of the coil, in Equation (3). The number of coil turns is proportional to the inductance strength.

Therefore, the output power of the EMG mainly depended on the magnetic flux density of the magnet and the inductance strength of the coil, and the output power was proportional to both. Compared to the conventional magnetic field pole alternation, the Halbach magnet array could effectively enhance the magnetic flux density by 1.4 times [37]. Based on the Halbach magnet array, the flux density of the magnet increased to 427 mT, measured by a Gaussmeter, and the resistance of the single coil was 543 Ω, measured by a multimeter (Figure S2). The inductance strength was 22 mH. According to the distribution of the magnetic field generated by the bar magnet, the combination of a double rectangular coil fit better with the magnetic field, which improved the cross-sectional area and space utilization of the coil, as shown in Figure S2. According to the magnetic flux density of the magnet array, coils were placed as shown in Figure 2d. The best performance of the EMG among three coil combinations (single coil, series coil, and parallel coil) was tested by a linear stepping machine (Figure S3). The oscilloscope was connected to the coil pin with a 100 M probe, and the linear stepping machine was set to slide at 3.5 Hz. After testing, the combination of series coils was found to have the best overall performance. As shown in Figure 2e, the peak open-circuit voltage output was about 13.2 V, and the peak short-circuit current was about 6.44 mA. The effective voltage was 2.36 V, and the effective current was 1.58 mA. In addition, the output results of a single excitation are shown in Figure 2f, where the resonant frequency of the electrical signal was above 20 Hz. Therefore, the combination of the series coil and the Halbach magnet array was selected to construct the electromagnetic power generation part.

### 3.3. Performance of MHP-HNG

**Figure 3:**
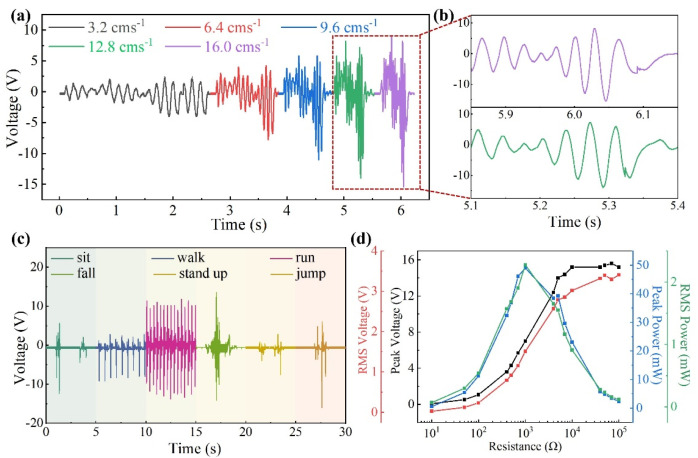
Performance characterization of the MHP-HNG. (**a**) Open-circuit voltage output of the wearable sensor of the servo motor at the speed of 3.2–16.0 cm/s. (**b**) Open-circuit voltage output magnification at 12.8 cm/s (green) and 16.0 cm/s (purple). (**c**) Comparison of the voltage signal output of the wearable sensor under different motion states of the human body. (**d**) Peak voltage, peak power, effective voltage, and effective power output of the sensor when externally loaded with different resistances.

Combining the above output characteristics, the performance of wearable sensors for generating triboelectric sensing signals during velocity changes was tested. Different speed test sequences (3.2 cm/s, 6.4 cm/s, 9.6 cm/s, 12.8 cm/s, and 16.0 cm/s) were designed to verify the effect of speed on the open-circuit voltage output, as shown in Figure S1. The results demonstrated that by increasing the sliding speed, the voltage amplitude increased from 4.0 V to 15.4 V (Figure 3a). The stability of triboelectric sensing signals was proven because the waveform of the signal output did not change significantly from a single traveling period at different sliding speeds. When the signal curves of 12.8 cm/s and 16.0 cm/s were amplified simultaneously, the signal frequency increased with the increase in speed (Figure 3b). The above experiments proved that voltage amplitude and signal frequency could be used as the basis for determining the speed excitation. However, compared with the mechanical fixed excitation, the magnitude and direction of the vibration excitation produced by humans are more random. When the mass block of the sensor vibrated vertically with respect to the shell, the maximum vibration energy was generated from the sensor at the chest position. Meanwhile, the minimum internal resistance suffered by the sensor which resulted in the highest conversion efficiency [38,39]. Therefore, the actual sensing performance of the sensor device by wearing it on the chest was tested. The oscilloscope recorded the open-circuit voltage outputs generated by sitting up, walking, running, falling, and other states (Figure 3c). When the human was in the jumping or running state, the wearable sensor would be excited by vibration, and the output voltage was significantly greater than that generated in other states. When the human accidentally fell, the mass block in the wearable sensor changed from the resting state to the high-frequency vibration state. The sensor responded to changes in acceleration and output an open-circuit voltage signal of 14.14 V. Therefore, the difference in voltage signal output was used as a judgement criterion for changes in human movement states.

The charging performance of the EMG was tested by a linear stepper at the frequency of 3.5 Hz, as shown in Figure S3. The results showed that the peak voltage and effective voltage increased with the increasing value of load resistance. When the resistor of 1 kΩ was connected, the wearable sensor had the maximum output power and effective power, which were 49 mW and 2.28 mW, respectively (Figure 3d). In addition, the charging performance of the wearable sensor was tested by using different capacitors with rectifiers, and the charging curve is shown in Figure S4. The supercapacitors of 1.0 mF, 2.2 mF, and 4.0 mF were charged to 2 V, 2.5 V, and 4.1 V in 10 s, respectively. Therefore, the EMG had a good charging performance, and the wearable sensor could effectively collect and use the electrical energy.

### 3.4. Application of MHP-HNG

#### 3.4.1. Application of Self-Powered Wireless Optical Communication Sensing System

**Figure 4:**
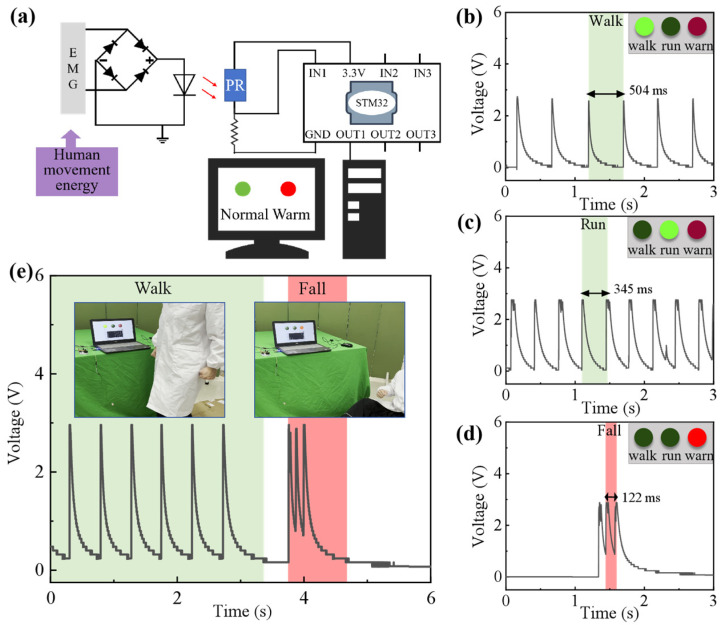
Application of a self-powered wireless optical communication sensing system. (**a**) Working principle of the self-powered wireless optical communication sensing system. Comparison of the output voltages of wearable sensors and the corresponding LabVIEW interfaces for the human body in walking (**b**), running (**c**), and falling (**d**) states. (**e**) Comparison of the output voltages of self-powered wireless optical communication sensing systems in monitoring walking and falling and the corresponding application scenes.

The optical communication system is a high-rate wireless information transmission system, which can provide reliable technical support for high-frequency electrical signal output generated by wearable sensors in response to human body movements. The optical communication sensing system uses the electromagnetic power generation signal of the wearable sensor to drive the light-emitting diode (LED) as the signal source. The voltage output from the sensor meets the threshold for lighting up LED with constant intensity. The system uses a photoresistor as the receiver to detect the change in luminosity and convert it to voltage change, which enables the transmission of the electrical signal from the sensor to the receiver. The specific schematic diagram is shown in Figure 4a. Therefore, the combination of optical communication technology and wearable sensors is expected to realize human health monitoring.

To verify the feasibility of optical communication sensing, the influence of certain factors on the transmission performance of the optical communication sensing system were explored, including the output frequency of the wearable sensor and the placement distance between the signal source and the receiver. As the placement distance decreased, the amplitude of the voltage signal generated by the receiver increased accordingly (Figure S5). Under different output frequencies of wearable sensors, the voltage drops in the receivers were basically same, as shown in Figure S6. The results showed that the magnitude of the voltage signal on the voltage drop of the receiver was inversely proportional to the distance between the signal source and the receiver. Meanwhile, the output of the LED was stable and not affected by the output voltage of the wearable sensor. Based on the above information, the wearable sensor and optical communication technology were used to build a wireless optical communication sensing system. The physical diagrams of the wireless optical communication sensing system are shown in Figure S7. First, wearable sensors collected the mechanical energy of human motion and converted it into electrical energy to drive LED. The photoresistor received the light signal that carried the energy information and converted it into an electrical signal. The single-chip microcomputer compared and processed the electrical signal information and sent the flag to the terminal, which could display the corresponding motion state. The clear LabVIEW interfaces are shown in Figure S8. When a person was walking and running, respectively, the peak voltage intervals were 504 ms and 345 ms, and the voltage frequencies were about 2 Hz and 2.9 Hz, as shown in Figure 4b,c. When a person had a fall accident, the voltage peak interval decreased to 122 ms and the frequency was about 8.2 Hz (Figure 4d). In the actual demonstration, when a person’s walking state changed to an accidental fall state, the interface immediately switched to a red warning light to alert the accident (Figure 4e). Based on the same-frequency vibration of the TENG and EMG in the wearable sensor, the wireless optical communication sensing system was able to effectively detect the human body’s movement states, which is of great significance for the development of self-powered sensing.

#### 3.4.2. Application of Self-Powered Wireless Communication Sensing System

**Figure 5:**
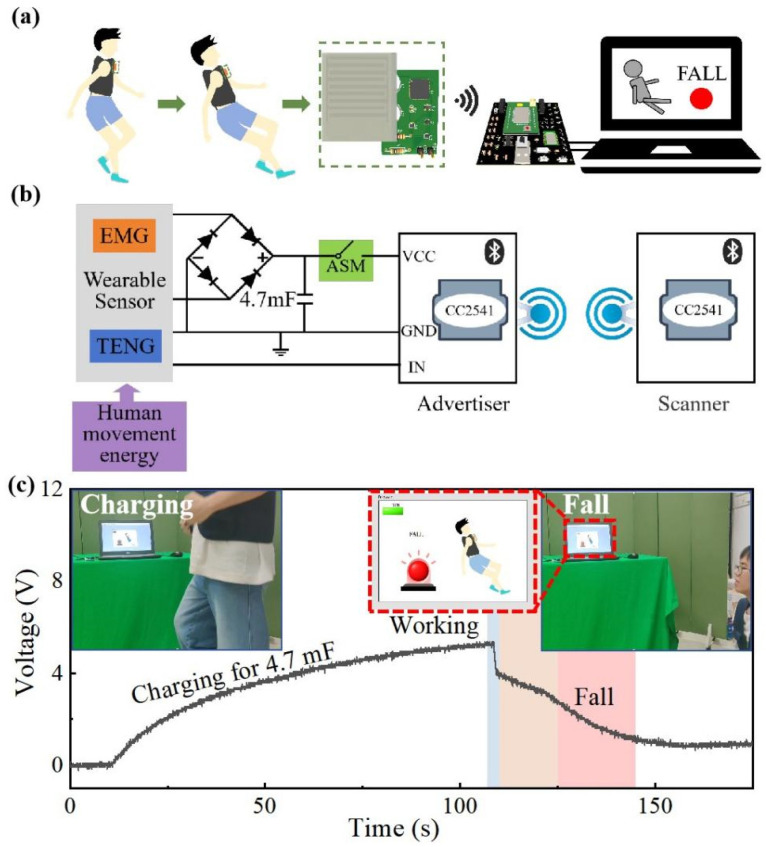
Application of a self-powered wireless communication sensing system. (**a**) Scenario diagram of the application scenario of a self-powered wireless communication sensing system. (**b**) The working principle of the self-powered wireless communication sensing system. (**c**) The change in the charge and discharge curve of the self-powered wireless communication sensor system, and the demonstration scene shown on the computer terminal interface of the normal motion and fall states of the human body. In this picture, the blue color represents the process of circuit conduction, the orange color represents the working state, the pink color represents the process of sending the signal.

The above results showed that the wireless optical communication system can recognize the change in the state of human motion, but it still requires an additional external power supply system to maintain operation. A reasonable circuit design was used to enable wearable sensors to achieve the integration of sensing and energy-harvesting functions, which allowed the wireless communication sensing system to realize self-powered wireless sensing of human motion states. The whole scene is shown in Figure 5a. The wearable sensor was bound to the person’s chest to continuously capture the mechanical vibration energy of human motion and convert it into an electromagnetic power generation signal to power the wireless communication sensing system. At the same time, motion information mapped from the triboelectric sensing signal was transmitted through the wireless communication sensing system. The automatic switching circuit module (ASM) and Bluetooth module were combined to design a self-powered wireless communication sensing system (Figure 5b). The whole circuit physical drawing is shown in Figure S9. When a person moves, the vibration energy is captured by the electromagnetic power generation part of the sensor and converted into alternate current (AC) electrical energy through the principle of electromagnetic induction. The ASM power management circuit receives the AC electrical energy generated by the sensor and converts it into direct current (DC) electrical energy through the rectifier circuit unit and charges the 4.7 mF capacitor. The electrical energy is stored in the capacitor, which realizes the self-powered function. At the same time, two cc2541 Bluetooth modules were used as the master and slave, respectively, to build the wireless communication system. When the capacitor is charged to the detection threshold voltage, the automatic switching module quickly turns on and releases the stored energy to maintain the operation of the Bluetooth module. After the Bluetooth module of the slave is operated, the internal analog-to-digital conversion (ADC) acquisition unit continuously collects the triboelectric sensing signals output by the wearable sensor. When a fall accident occurs, a clear difference between the sensing signal of the fall and normal movement state will be perceived by the system. Then, it will send an alert to realize the purpose of intelligent perception and real-time monitoring of human motion.

The wearable sensor was tested in the practical medical monitoring scene based on the sensing and self-powered properties that wearable sensors possess. Figure 5c shows the charging and discharging curve of the system and the terminal display interface in different motion states in the practical application. The entire self-powered wireless sensing system was worn on the human body. After 130 s of charging, the voltage of the 4.7 mF capacitor in the ASM module was charged to 4 V. At this time, the wireless communication sensing system entered the normal operation state. When a person fell, the Bluetooth module detected a change in the output voltage amplitude and immediately sent the broadcast packet. At the same time, a warning light was illuminated at the display terminal to provide early warning. After waking up the Bluetooth module, the wireless communication sensing system monitored the motion status information of the human motion in real time for more than 35 s. Based on the above application demonstration of practical scenes, the self-powered wireless communication sensing system provided a practical basis for remote monitoring in the medical field.

## 4. Conclusions

In conclusion, a wearable sensor coupling the TENG and EMG for harvesting mechanical vibration energy and monitoring human motion states was comprehensively proposed. By combining the kinematic properties of flexible substrates and nanogenerators, the TENG and EMG could achieve the same-frequency vibration and simultaneous output. Optimization of the friction layer, magnet, and coil structure enabled the wearable sensor to achieve the sensing signal output of 14.14 V and the power output of 49 mW. Furthermore, a self-powered wireless communication sensing system combined with wireless communication technology was designed, which could realize real-time monitoring of the human movement status without an external power supply. Therefore, the wearable sensor is expected to achieve health monitoring, emergency warning, and rehabilitation assistance, which might further expand its application in the medical field.

## Data Availability

The data presented in this study are available in this article.

## Supplementary Materials

The following supporting information can be downloaded at: https://www.mdpi.com/article/10.3390/bios14080361/s1. Table S1: Characteristics comparison of the reported sensors with the MHP-HNG for health monitoring. Figure S1: Optimizing the width of the grid structure and testing the sensing performance of the TENG based on a servo motor. Figure S2: The magnetic field strength of the Halbach magnet array, the physical diagram of the combination of the double rectangular coils, and the resistance of the single coil. Figure S3: Optimizing the coil combination mode and testing the charging performance of the EMG based on a linear stepper test platform. Figure S4: Charging curves within 10 s for the capacitors of 1.0 mF, 2.2 mF, and 4.0 mF. Figure S5: Exploring the effect of the placement distance between the signal source and the receiver on the voltage output of the voltage drop of the receiver. Figure S6: Exploring the effect of the output frequency of the wearable sensor on the voltage drop of the receiver. Figure S7: The circuit physical diagram of the wireless optical sensing system. Figure S8: The clear LabVIEW interfaces for the human body in walking (left), running (middle), and falling (right) states. Figure S9: The circuit physical diagram of the wireless sensing system.

## References

[1] Wu Z.Y., Zhang B.B., Zou H.Y., Lin Z.M., Liu G.L., Wang Z.L. (2019). Multifunctional Sensor Based on Translational-Rotary Triboelectric Nanogenerator. Adv. Energy Mater..

[2] Maharjan P., Bhatta T., Salauddin Rasel M., Salauddin M., Toyabur Rahman M., Park J.Y. (2019). High-Performance Cycloid Inspired Wearable Electromagnetic Energy Harvester for Scavenging Human Motion Energy. Appl. Energy.

[3] Mahmud M.A.P., Huda N., Farjana S.H., Asadnia M., Lang C. (2018). Recent Advances in Nanogenerator-Driven Self-Powered Implantable Biomedical Devices. Adv. Energy Mater..

[4] Tan P.C., Zheng Q., Zou Y., Shi B.J., Jiang D.J., Qu X., Ouyang H., Zhao C., Cao Y., Fan Y. (2019). A Battery-Like Self-Charge Universal Module for Motional Energy Harvest. Adv. Energy Mater..

[5] Gao Y., Yu L., Yeo J.C., Lim C.T. (2020). Flexible Hybrid Sensors for Health Monitoring: Materials and Mechanisms to Render Wearability. Adv. Mater..

[6] Liu F., Liu K., Rafique S., Xu Z., Niu W., Li X., Wang Y., Deng L., Wang J., Yue X. (2023). Highly Efficient and Stable Self-Powered Mixed Tin-Lead Perovskite Photodetector Used in Remote Wearable Health Monitoring Technology. Adv. Sci..

[7] World Health Organization (2017). Who Guidelines Approved by the Guidelines Review Committee. Integrated Care for Older People: Guidelines on Community-Level Interventions to Manage Declines in Intrinsic Capacity.

[8] Chan L.L.Y., Arbona C.H., Brodie M.A., Lord S.R. (2023). Prediction of Injurious Falls in Older Adults Using Digital Gait Biomarkers Extracted from Large-Scale Wrist Sensor Data. Age Ageing.

[9] Warmerdam E., Hausdorff J.M., Atrsaei A., Zhou Y., Mirelman A., Aminian K., Espay A.J., Hansen C., Evers L.J., Keller A. (2020). Long-Term Unsupervised Mobility Assessment in Movement Disorders. Lancet Neurol..

[10] Liu X., Sun C., Guo Z., Xia X., Jiang Q., Ye X., Shang J., Zhang Y., Zhu X., Li R.W. (2023). Near-Sensor Reservoir Computing for Gait Recognition Via a Multi-Gate Electrolyte-Gated Transistor. Adv. Sci..

[11] Zhu Z., Estevez D., Feng T., Chen Y., Li Y., Wei H., Wang Y., Wang Y., Zhao L., Jawed S.A. (2024). A Novel Induction-Type Pressure Sensor Based on Magneto-Stress Impedance and Magnetoelastic Coupling Effect for Monitoring Hand Rehabilitation. Small.

[12] Wang C., Lai S.K., Wang Z.C., Wang J.M., Yang W.Q., Ni Y.Q. (2019). A Low-Frequency, Broadband and Tri-Hybrid Energy Harvester with Septuple-Stable Nonlinearity-Enhanced Mechanical Frequency Up-Conversion Mechanism for Powering Portable Electronics. Nano Energy.

[13] He J., Fan X.M., Zhao D.Y., Cui M., Han B., Hou X., Chou X. (2022). A High-Efficient Triboelectric-Electromagnetic Hybrid Nanogenerator for Vibration Energy Harvesting and Wireless Monitoring. Sci. China Inf. Sci..

[14] Salauddin M., Rasel M.S., Kim J.W., Park J.Y. (2017). Design and Experiment of Hybridized Electromagnetic-Triboelectric Energy Harvester Using Halbach Magnet Array from Handshaking Vibration. Energy Convers. Manag..

[15] Salauddin M., Toyabur R.M., Maharjan P., Park J.Y. (2018). High Performance Human-Induced Vibration Driven Hybrid Energy Harvester for Powering Portable Electronics. Nano Energy.

[16] Zhang Z.X., He J., Wen T., Zhai C., Han J.Q., Mu J., Jia W., Zhang B., Zhang W., Chou X. (2017). Magnetically Levitated-Triboelectric Nanogenerator as a Self-Powered Vibration Monitoring Sensor. Nano Energy.

[17] Tao X., Zhou Y., Qi K., Guo C., Dai Y., He J., Dai Z. (2022). Wearable Textile Triboelectric Generator Based on Nanofiber Core-Spun Yarn Coupled with Electret Effect. J. Colloid Interface Sci..

[18] Gu Y., Liu W., Zhao C., Wang P. (2020). A Goblet-Like Non-Linear Electromagnetic Generator for Planar Multi-Directional Vibration Energy Harvesting. Appl. Energy.

[19] Yun J., Patel S.N., Reynolds M.S., Abowd G.D. (2011). Design and Performance of an Optimal Inertial Power Harvester for Human-Powered. IEEE Trans. Mob. Comput..

[20] Salauddin M., Toyabur R.M., Maharjan P., Rasel M.S., Kim J.W., Cho H., Park J.Y. (2018). Miniaturized Springless Hybrid Nanogenerator for Powering Portable and Wearable Electronic Devices from Human-Body-Induced Vibration. Nano Energy.

[21] Kim J.W., Salauddin M., Cho H., Rasel M.S., Park J.Y. (2019). Electromagnetic Energy Harvester Based on a Finger Trigger Rotational Gear Module and an Array of Disc Halbach Magnets. Appl. Energy.

[22] Rahman M.T., Rana S.M.S., Salauddin M., Maharjan P., Bhatta T., Kim H., Cho H., Park J.Y. (2020). A Highly Miniaturized Freestanding Kinetic-Impact-Based Non -Resonant Hybridized Electromagnetic-Triboelectric Nanogenerator for Human Induced Vibrations Harvesting. Appl. Energy.

[23] Deng H.T., Wang Z.Y., Wang Y.L., Wen D.L., Zhang X.S. (2022). Integrated Hybrid Sensing and Microenergy for Compact Active Microsystems. Microsyst. Nanoeng..

[24] Tang G., Wang Z., Hu X., Wu S.J., Xu B., Li Z., Yan X., Xu F., Yuan D., Li P. (2022). A Non-Resonant Piezoelectric-Electromagnetic-Triboelectric Hybrid Energy Harvester for Low-Frequency Human Motions. Nanomaterials.

[25] Salauddin M., Toyabur R.M., Maharjan P., Rasel M.S., Cho H., Park J.Y. (2019). Design and Experimental Analysis of a Low-Frequency Resonant Hybridized Nanogenerator with a Wide Bandwidth and High Output Power Density. Nano Energy.

[26] Wang P.H., Liu R.Y., Ding W.B., Zhang P., Pan L., Dai G., Zou H., Dong K., Xu C., Wang Z.L. (2018). Complementary Electromagnetic-Triboelectric Active Sensor for Detecting Multiple Mechanical Triggering. Adv. Funct. Mater..

[27] Wu Z.Y., Ding W.B., Dai Y.J., Dong K., Wu C.S., Zhang L., Lin Z., Cheng J., Wang Z.L. (2018). Self-Powered Multifunctional Motion Sensor a, Enabled by Magnetic-Regulated Triboelectric Nanogenerator. ACS Nano.

[28] Maharjan P., Bhatta T., Cho H., Hui X., Park C., Yoon S., Salauddin M., Rahman M.T., Rana S.S., Park J.Y. (2020). A Fully Functional Universal Self-Chargeable Power Module for Portable/Wearable Electronics and Self-Powered Iot Applications. Adv. Energy Mater..

[29] Rana S.M.S., Salauddin M., Sharifuzzaman M., Lee S.H., Shin Y.D., Song H., Jeong S.H., Bhatta T., Shrestha K., Park J.Y. (2022). Ultrahigh-Output Triboelectric and Electromagnetic Hybrid Generator for Self-Powered Smart Electronics and Biomedical Applications. Adv. Energy Mater..

[30] Rana S.M.S., Rahman M.T., Salauddin M., Maharjan P., Bhatta T., Cho H., Park J.Y. (2020). A Human-Machine Interactive Hybridized Biomechanical Nanogenerator as a Self-Sustainable Power Source for Multifunctional Smart Electronics Applications. Nano Energy.

[31] Chen Y.L., Liu D., Wang S., Li Y.F., Zhang X.S. (2019). Self-Powered Smart Active RFID Tag Integrated with Wearable Hybrid Nanogenerator. Nano Energy.

[32] Rahman M.T., Rana S.M.S., Salauddin M., Maharjan P., Bhatta T., Park J.Y. (2020). Biomechanical Energy-Driven Hybridized Generator as a Universal Portable Power Source for Smart/Wearable Electronics. Adv. Energy Mater..

[33] Iqbal M., Nauman M.M., Khan F.U., Abas E., Cheok Q., Aissa B. (2021). Nonlinear Multi-Mode Electromagnetic Insole Energy Harvester for Human-Powered Body Monitoring Sensors: Design, Modeling, and Characterization. Proc IMechE Part C J. Mech. Eng. Sci..

[34] Pu X., Li L.X., Song H.Q., Du C.H., Zhao Z.F., Jiang C., Cao G., Hu W., Wang Z.L. (2015). A Self-Charging Power Unit by Integration of a Textile Triboelectric Nanogenerator and a Flexible Lithium-Ion Battery for Wearable Electronics. Adv. Mater..

[35] Yi F., Zhang Z., Kang Z., Liao Q.L., Zhang Y. (2019). Recent Advances in Triboelectric Nanogenerator-Based Health Monitoring. Adv. Funct. Mater..

[36] Li T., Xu Y., Willander M., Xing F., Cao X., Wang N., Wang Z.L. (2016). Lightweight Triboelectric Nanogenerator for Energy Harvesting and Sensing Tiny Mechanical Motion. Adv. Funct. Mater..

[37] Zhu D.B., Beeby S., Tudor J., Harris N. (2012). Vibration Energy Harvesting Using the Halbach Array. Smart Mater. Struct..

[38] Fuentes-Abolafio I.J., Trinidad-Fernández M., Escriche-Escuder A., Roldán-Jiménez C., Arjona-Caballero J.M., Bernal-López M.R., Ricci M., Gómez-Huelgas R., Pérez-Belmonte L.M., Cuesta-Vargas A.I. (2022). Kinematic Parameters That Can Discriminate in Levels of Functionality in the Six-Minute Walk Test in Patients with Heart Failure with a Preserved Ejection Fraction. J. Clin. Med..

[39] Lin H.C., Chen M.J., Lee C.H., Kung L.C., Huang J.T. (2023). Fall Recognition Based on an IMU Wearable Device and Fall Verification through a Smart Speaker and the loT. Sensors.

