# Supplementary material for: Wearable Sensors Based on Miniaturized High-Performance Hybrid Nanogenerator for Medical Health Monitoring
Source: Biosensors (Basel). 2024 Jul 25;14(8):361. doi: 10.3390/bios14080361 (PMC11352411; doi:10.3390/bios14080361)
Supplement: Supplementary file 1 [file biosensors-14-00361-s001.zip › biosensors-3025239-supplementary.pdf]

Section S1. A brief overview of the characteristics of sensors based on TENG, EMG and TENG-EMG hybrid nanogenerators for health monitoring.

Table S1. Characteristics comparison of the reported sensors with MHP-HNG for health monitoring.

Mechanism	Dimension (cm^3)	Voltage (V)	Power (mW)	Advantages	Weaknesses	Application	Reference
EMG	47.8	0.17	0.839	Low internal impedances<br>High current	Low voltage output	Energy harvesters installed into the shoe	[1]
TENG	25.0	50	-	High voltage output	High internal impedances<br>Low current	Powering a heartbeat meter strap capable of remote communication with a smart phone	[2]
TENG-EMG hybrid	45.9	13.15	144.1	High power output	Large size and weight	Portable energy harvesters to sustainably power portable/wearable devices in extreme situations	[3]
	24.0	14.14	49	High power output<br>More comfortable	-	A wearable sensor for monitoring human falls with wireless communication sensing	This work (MHP-HNG)

Section S2. The structural optimization and performance characterization of the TENG and EMG are verified by the test platform. A Gaussmeter is used to measure the magnetic field strength and a multimeter is used to measure the electrical resistance as the basis for performance characterization. The voltage amplitude of the charging curve demonstrates the charging capability of the EMG. This section demonstrates the optimization and performance of MHP-HNG.

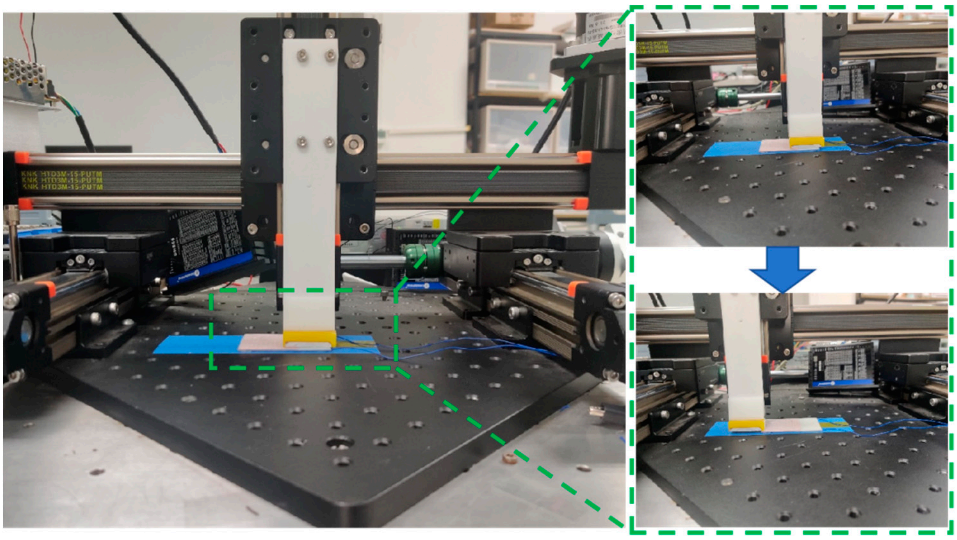

Figure S1. Optimizing the width of the grid structure and testing the sensing performance of TENG based on a servo motor.

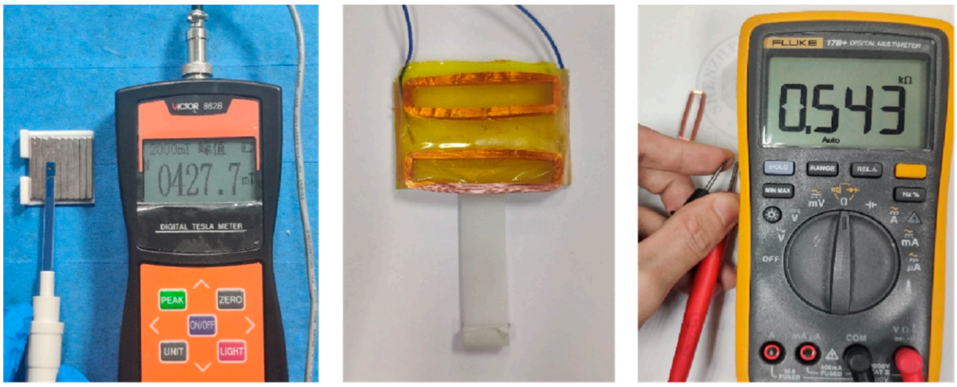

Figure S2. The magnetic field strength of the Halbach magnet array, the physical diagram of the combination of the double rectangular coils, and the resistance of the single coil.

mF.

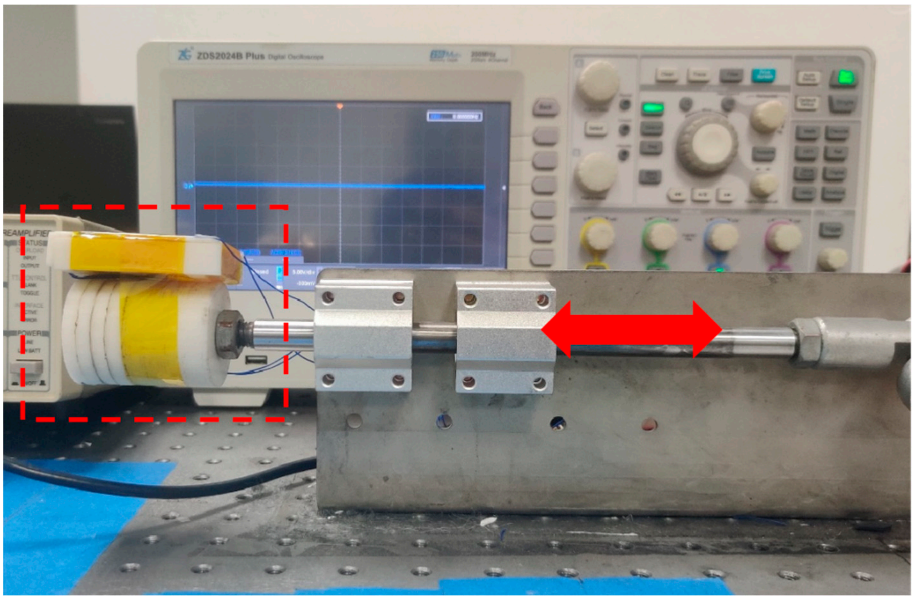

Figure S3. Optimizing the coil combination mode and testing the charging performance of EMG based on a linear stepper test platform.

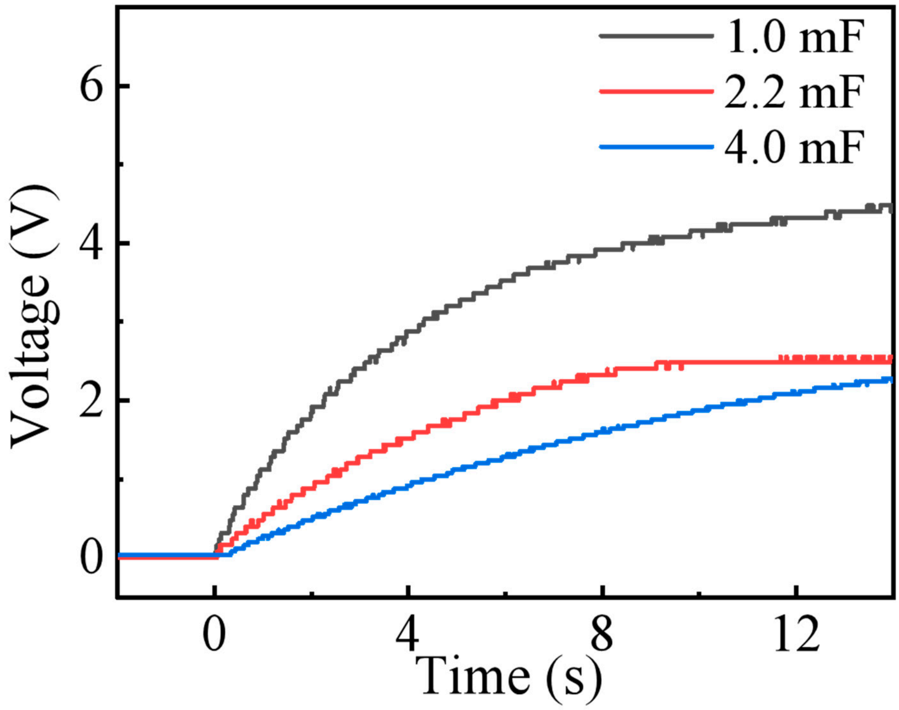

Figure S4. Charging curves within 10 s for the capacitors of 1.0 mF, 2.2 mF, and 4.0 mF.

Section 3. Optical communication system is a high-rate wireless information transmission system, which can provide reliable technical support for wearable sensors in response to human body movements. This section demonstrates the performance of the wireless optical communication sensing system.

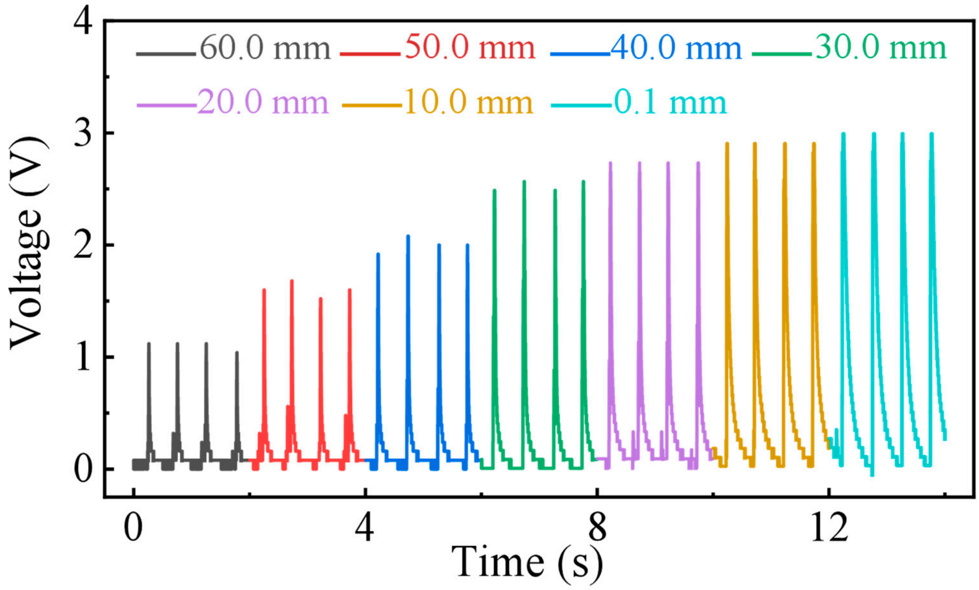

Figure S5. Exploring the effect of the placement distance between the signal source and the receiver on the voltage drop of the receiver.

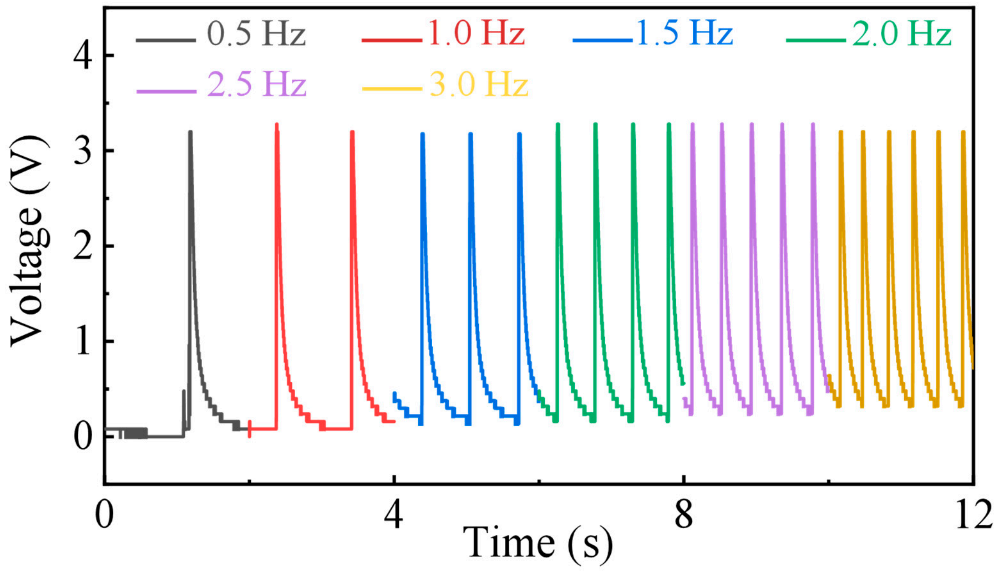

Figure S6. Exploring the effect of the output frequency of the wearable sensor on the voltage drop of the receiver.

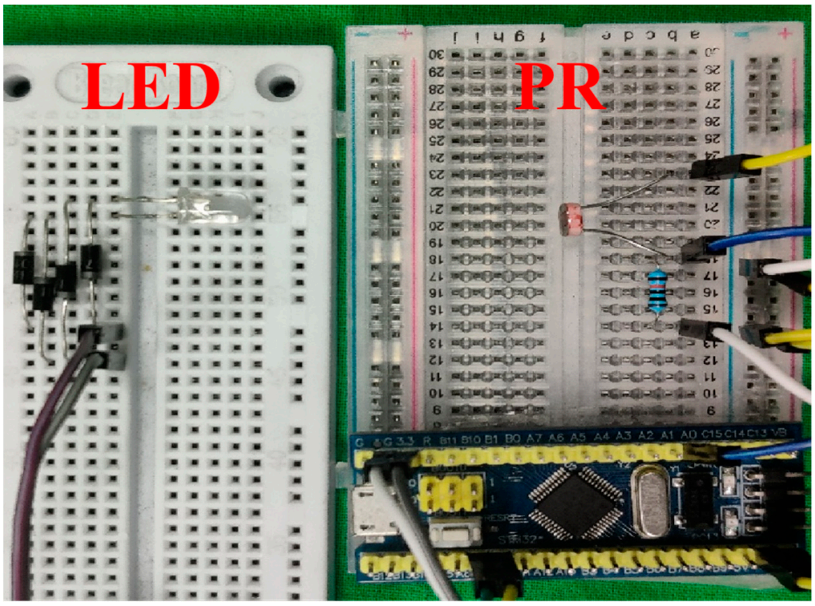

Figure S7. The circuit physical diagram of the wireless optical sensing system.

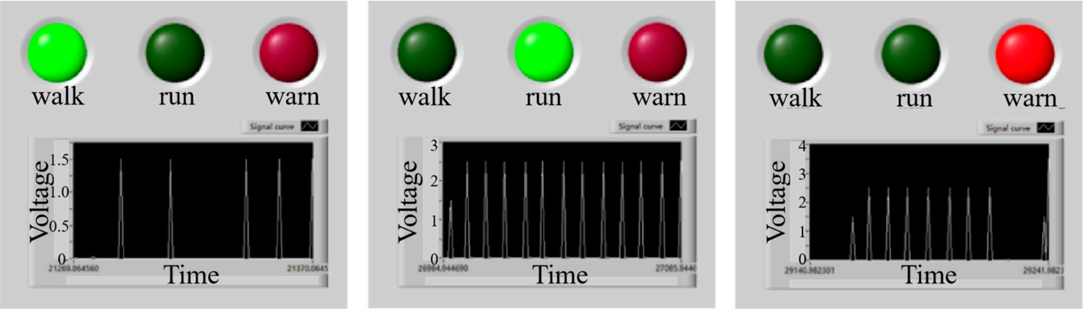

Figure S8. The clear LabVIEW interfaces for the human body in walking (left), running (middle), and falling (right) states.

Section 4. The wireless communication sensing system combines wearable sensors and Bluetooth modules to realize wireless remote monitoring. This section shows a physical diagram of the wireless sensing system. In the practical application, PI adhesive is sealed outside the shell to increase the dryness and sealing of the sensor.

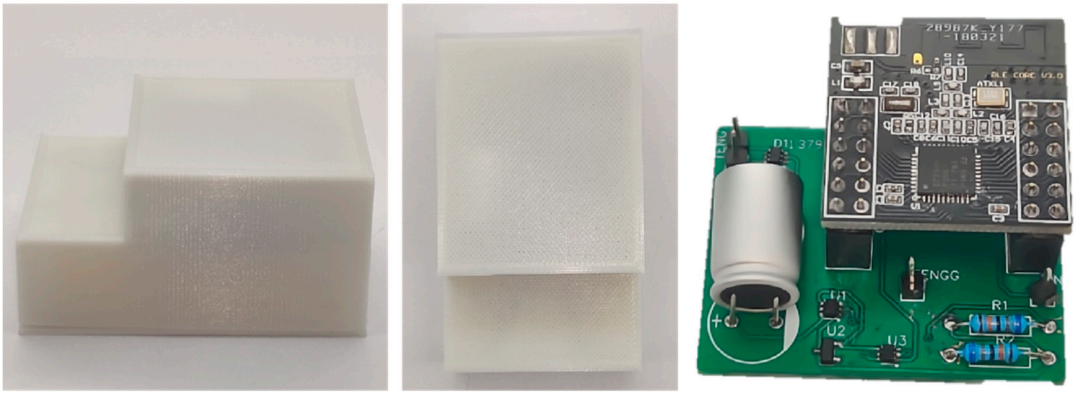

Figure S8. Package and circuit physical drawings of the wireless sensing system.

References

1. Iqbal, M.; Nauman, M.M.; Khan, F.U.; Abas, E.; Cheok, Q., et al. Nonlinear Multi-Mode Electromagnetic Insole Energy Harvester for Human-Powered Body Monitoring Sensors: Design, Modeling, and Characterization. *Proc IMechE Part C:J Mechanical Engineering Science* **2021**, *235*, 6415-6426.
2. Pu, X.; Li, L.X.; Song, H.Q.; Du, C.H.; Zhao, Z.F., et al. A Self-Charging Power Unit by Integration of a Textile Triboelectric Nanogenerator and a Flexible Lithium-Ion Battery for Wearable Electronics. *Adv. Mater.* **2015**, *27*, 2472-2478.
3. Rahman, M.T.; Rana, S.M.S.; Salauddin, M.; Maharjan, P.; Bhatta, T., et al. Biomechanical Energy-Driven Hybridized Generator as a Universal Portable Power Source for Smart/Wearable Electronics. *Adv. Energy Mater.* **2020**, *10*, 1903663.
